# Supplementary material for: The interactome of intact mitochondria by cross-linking mass spectrometry provides evidence for coexisting respiratory supercomplexes
Source: Mol Cell Proteomics. 2017 Dec 8;17(2):216–32. doi: 10.1074/mcp.RA117.000470 (PMC5795388; doi:10.1074/mcp.RA117.000470)
Supplement: Supplemental Data [file supp_RA117.000470_133922_0_supp_23978_fzffwf.zip › spectra_annotation/mito_DR_spectra_annotation/398-1-3-1-11-1.pdf]

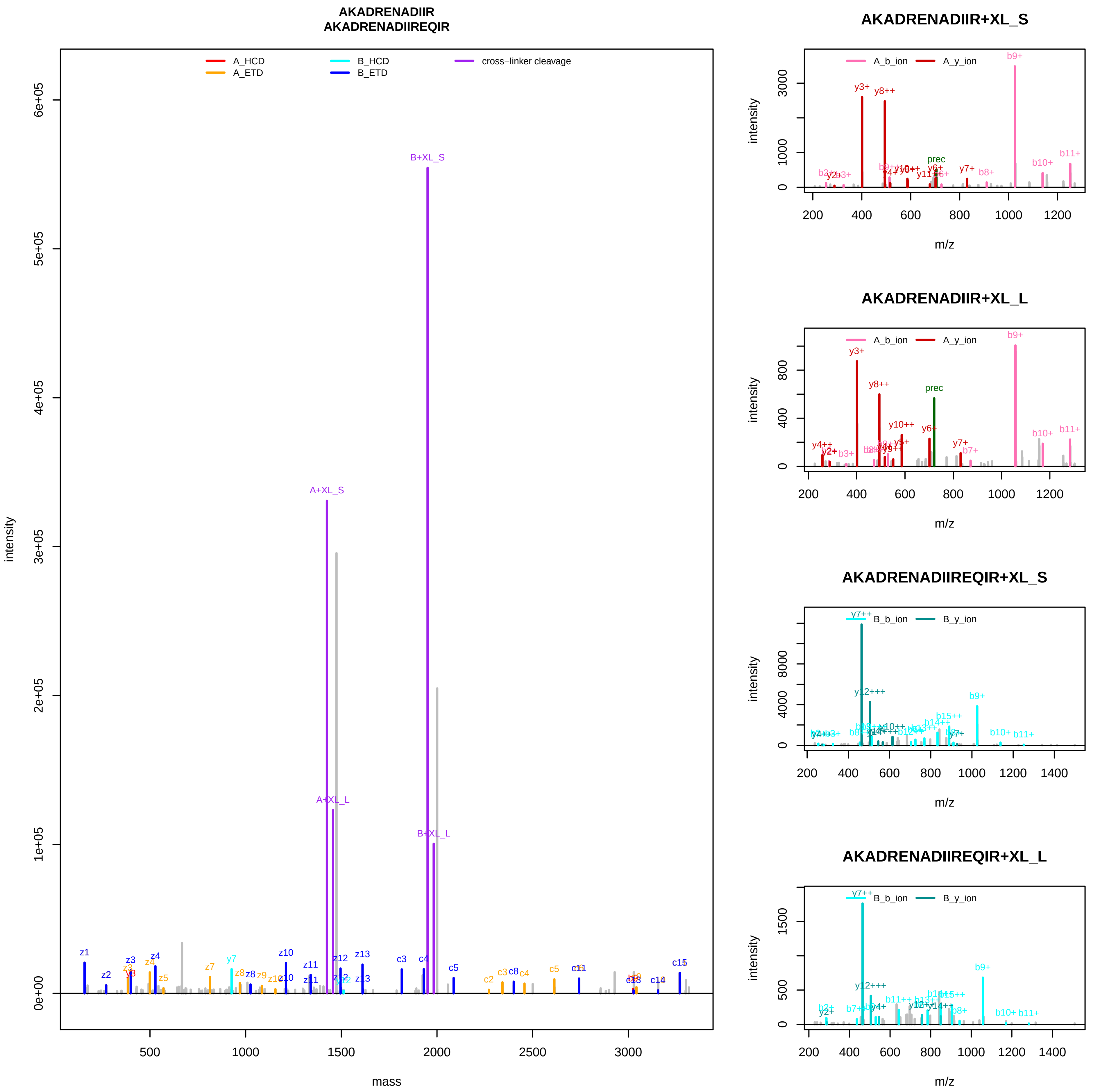
AKADRENADIIR
AKADRENADIIREQIR
A_HCD
A_ETD
B_HCD
B_ETD
cross−linker cleavage
B+XL_S
A+XL_S
A+XL_L
B+XL_L
intensity
mass
6e+05
5e+05
4e+05
3e+05
2e+05
1e+05
0e+00
500
1000
1500
2000
2500
3000
AKADRENADIIR+XL_S
A_b_ion
A_y_ion
b9+
y3+
y8++
prec
y7+
b10+
b11+
intensity
m/z
AKADRENADIIR+XL_L
A_b_ion
A_y_ion
b9+
y3+
y8++
prec
y10++
y6+
y7+
b10+
b11+
intensity
m/z
AKADRENADIIREQIR+XL_S
B_b_ion
B_y_ion
y7++
y12+++
b9+
b15++
b14++
b10+
b11+
intensity
m/z
AKADRENADIIREQIR+XL_L
B_b_ion
B_y_ion
y7++
y12+++
b9+
b10+
b11+
intensity
m/z
